# A comparison of transforaminal lumbar interbody fusion (TLIF) cage material on fusion rates: A systematic review and network meta-analysis

**DOI:** 10.1016/j.wnsx.2024.100392

**Published:** 2024-05-25

**Authors:** Sutipat Pairojboriboon, Supranee Niruthisard, Chandhanarat Chandhanayingyong, Chalinee Monsereenusorn, Siwaporn Poopan, Sheng-Fu Larry Lo

**Affiliations:** a Department of Orthopaedic Surgery, Phramongkutklao Hospital and Phramongkutklao College of Medicine, Bangkok, Thailand; b Department of Anesthesiology, King Chulalongkorn Memorial Hospital, Bangkok, Thailand; c Department of Orthopaedic Surgery, Faculty of Medicine, Siriraj Hospital, Mahidol University, Bangkok, Thailand; d Department of Pediatrics, Phramongkutklao Hospital and Phramongkutklao College of Medicine, Bangkok, Thailand; e Faculty of Social Sciences and Humanities, Mahidol University, Thailand; f Deparment of Neurosurgery, Zucker School of Medicine at Hofstra/Northwell, New York, USA

**Keywords:** 3D printing, TLIF, Lumbar interbody fusion, PEEK, Titanium, Material, Fusion rate

## Abstract

**Background:**

A wide variety of materials are used for lumbar interbody fusion, but there is no unified consensus on the superiority of one material over another. The aim of this systematic review and network meta-analysis (NMA) is to compare and rank the various TLIF interbody materials based on fusion rates.

**Methods:**

We queried PubMed, EMBASE and Scopus from inception until August 2023, in which 2135 studies were identified. Inclusion criteria were applied based on the PRISMA guidelines. The fusion assessment employed the Bridwell's criteria with a length of follow-up of at least 12 months. The NMA was conducted to compare multiple approaches from multiple studies using the frequentist framework with STATA16.

**Results:**

In total, 13 TLIF studies involving 1919 patients with 1981 lumbar interbody levels fulfilled our eligibility criteria. Seven different cage materials were utilized: polyetheretherketone (PEEK, as the reference), allograft, autograft, PEEK with titanium coating (TiPEEK), titanium, carbon/carbon fiber reinforced polymer (CFRP) and 3D-printed titanium. The average patient age was 60.9 (*SD* = 7.5) years old. When compared to PEEK, the other six materials did not have a significantly different rate of lumbar fusion. However, the SUCRA number of the 3D-printed titanium, TiPEEK, Ti, allograft, autograft, CFRP, and PEEK were 0.8, 0.6, 0.5, 0.5, 0.4, 0.4, and 0.3 consecutively.

**Conclusions:**

Based on a network meta-analysis within the confines of our clinical study, 3D-printed titanium interbody cage may promote the highest success rate of fusion while PEEK may be the material with the least success rate of fusion in TLIF.

## 1 Introduction

Transforaminal lumbar interbody fusion (TLIF) was first reported by Harms and Rolinger in 1982.^1^ It has become popular among spine surgeons because the procedure is safe and effective.^2^^,^^3^ Although the overall lumbar interbody fusion rates for TLIF are high (above 89 %),^4^^,^^5^ failed TLIF cage could generate pain and disability requiring revision surgery.^6^ The results of revision procedures have been relatively poor, with only 30–50 % rate of functional success.^7^ Careful surgical strategies including interbody material and optimal graft selection are important to achieve the highest fusion rate.

Various types of interbody material are available. Each has both advantages and disadvantages over the others. The most predominant used spacers are titanium and polyetheretherketone (PEEK) because they are biocompatible and able to provide the mechanical stability.^8^ The other options to PEEK are CFRP, titanium-coated PEEK, femoral strut allograft, morselized local autograft, as well as hydroxyapatite-polymer composites and coatings. Although spine interbody implants are commonly used, the optimal material selection remains controversial with few clinical studies comparing fusion rates between interbody materials.

To the best or our knowledge, there has been only one report of standard pairwise meta-analyses on role of titanium (Ti) and PEEK cages which have shown no significant difference in terms of lumbar fusion rates.^9^ Several studies have evaluated the role of interbody material in cervical spinal fusion, which cannot be used to interpret in our study.^10^, ^11^, ^12^ This study aimed to compare the fusion rates between different types of interbody materials used for TLIF. Using the network meta-analysis (NMA), we can identify and rank the interbody materials based on the lumbar interbody fusion rates when using TLIF procedures.

## 2 Methods

This study constitutes a systematic review with a NMA. We performed the method and reporting structure following the Preferred Reporting Items for Systematic Reviews and Meta-analyses (PRISMA) guidelines for NMA.

### 2.1 Search strategy and evidence selection

We queried PubMed, EMBASE, Scopus and Web of Science for relevant studies that matched our criteria from inception until August 2023. The search strategy consisted of keywords: “lumbar interbody fusion” AND “cage” with materials documentation “polyetheretherketone” OR “PEEK” OR “titanium” OR “carbon” OR “allograft” OR “autograft” OR “3D printing” OR “tantalum”. Only studies with full English-language text availability were considered. Two independent reviewers (SP1 and SP2) performed the two-stage screening for study relevance, first by title and abstract, and then, by the full text article review. A third senior author (SN) made a final judgment for any disagreement between the reviewers during the evidence selection process. The critical appraisal of our eligible articles was independently assessed and extracted by two authors (SP1 and SP2). using a standardized data extraction form including study design, sample size, patient characteristics i.e., age and sex, TLIF cage materials, types of bone graft, TLIF surgical levels, fusion assessment methods, clinical and radiographic outcomes and follow-up time. The risk of bias assessment for the nonrandomized trials was evaluated using the Newcastle-Ottawa Scale.^13^

### 2.2 Inclusion criteria

Eligibility criteria for our study included^1^: a diagnosis of degenerative lumbosacral spine disease^2^; a comparison between materials of Ti, PEEK, Ti-coated PEEK, allograft, autograft, CFRP, tantalum and 3D-printed Ti and^3^ a patient population of adult patients with more than 12 months of postoperative follow-up. Exclusion criteria included^1^: case reports, reviews, in vitro biomechanical studies, in vivo animal studies and computer modeling studies^2^; a diagnosis of idiopathic scoliosis, spinal infection, spinal oncology, autoimmune disease, and/or trauma^3^; surgical interventions of spondylectomy, corpectomy and corrective osteotomy procedures^4^; unspecified or uncommon interbody material^5^; expandable cage and^6^ use of bone morphogenic protein (BMP).

The rate of lumbar interbody fusion was the primary outcome. Lumbar fusion was evaluated using computed tomography (CT) scan or radiographic x-ray with a length of follow-up of at least 12 months. The criteria for fusion were defined as complete trabecular bridging of grafted bone material with or without a visual gap or grade I or II based on Bridwell's criteria.^14^^,^^15^ Grade I is defined as fusion with remodeling and trabeculae present; grade II is defined as an intact graft, but not fully remodeled and incorporated without lucencies. Some studies also defined nonunion as greater than 4° of motion on dynamic imaging.

### 2.3 Data synthesis and analysis

The direct evidence (head-to-head trials) were analyzed using a random-effect model and reported coefficient, standard error, *p*-value, and 95 % confidence interval (CI). The odds ratio (OR) with 95 % confidence intervals (CIs) were reported in the forest plot. The positive coefficient means OR greater than one. On the other hand, a negative coefficient means OR less than one. The statistical analyses in this study were performed by the biostatistician (SP2) using Stata (release 16, Stata- Corp LLC, TX, USA). The ranking probability of each TLIF cage material was calculated. This was achieved by calculating the log odds ratio for each type of interbody materials compared with the reference, namely, PEEK. The surface under the cumulative ranking (SUCRA) was also created to represent the cumulative probability for each interbody material to demonstrate the most desirable material option to gain the highest success fusion rate. The rank is presented both graphically and numerically for comparison in which the graph presents the area under the curve to indicate the probability of each material option. The numeric presentation offers overall ranking ranging from 0 to 100 %; a ranking closer to 100 % indicates a higher likelihood of that material option to be in the top rank, while a ranking closer to 0 provides a material option of lower likelihood of the bottom rank, than other options. We further analyzed the quality of the included studies for discrepancies using the NewCastle-Ottawa Scale (NOS).

## 3 Results

**Fig. 1:**
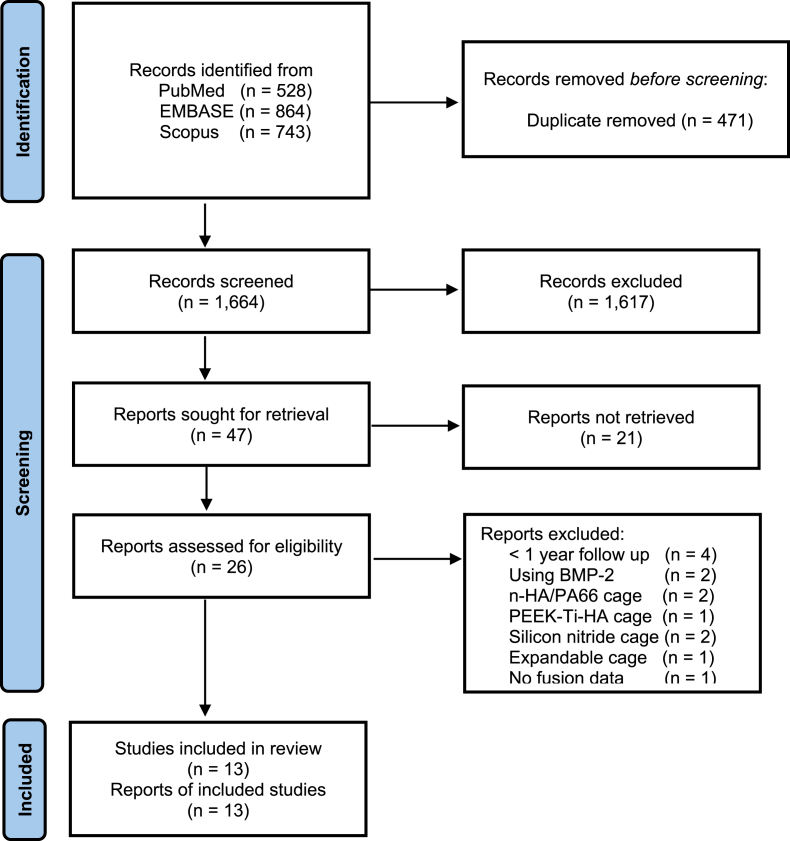
The PRISMA flow diagram of the include studies.

We identified 2135 articles: 528 from PubMed, 864 from EMBASE and 743 from Scopus (Fig. 1). In all, 471 were duplicated references; and the remaining 1664 articles underwent title and abstract review, of which 26 proved potentially eligible for our study and underwent full text review. Of these 26 TLIF studies with head-to-head comparisons of interbody materials, 13 studies were excluded (five studies using uncommon materials (n-HA/PA66, Silicon nitride, PEEK-Ti-HA), four studies with follow-up time less than one year, two studies using BMP, one study using expandable cage and one study without report of fusion rate).

A total of 13 TLIF studies were included in the NMA (Fig. 1). These studies included eight retrospective studies, three prospective studies and two randomized controlled trial with 1919 patients performed on 1981 lumbar interbody levels (Table 1). The study included 845 males and 1074 females with the average age of 60.9 years (*SD* = 7.5). The systematic review of the comparison of the different interbody materials is shown in Table 2. The fusion rate of each interbody material was PEEK (90.2 %), Ti (89.2 %), CFRP (100 %), allograft (96.5 %), TiPEEK (94.4 %), autograft (94.8 %) and 3D-printed Ti (90.9 %).

**Table 1:** Characteristics of the included studies.

Authors	Design	Average age	Male/Female	Materials	Autograft	Synthetic graft	L1-2	L2-3	L3-4	L4-5	L5-S1
Cutler 2006(47)	R	45.8	9/12	Allograft	21	21 (DBM)					
		50.2	8/10	PEEK	18	18 (DBM)					
Lv 2015(48)	R	51	32/52	PEEK	84					61	23
		53	37/59	Autograft						67	29
Mura 2011(49)	R	54.2	M = 62	PEEK	56						
		54.2	F = 38	CFRP	58						
Nemoto 2014(50)	R	40.7	22/1	Titanium	23					7	16
		42.9	23/2	PEEK	25					10	15
Rickert 2017(51)	RCT	67.7	6/14	Ti PEEK	22	22 (HA + B-TCP)		1	9	14	
		68.3	6/14	PEEK	20	20 (HA + B-TCP)		1	10	15	
Vazifehdan 2019(52)	R	71	M = 167	PEEK							
		71	F = 252	Titanium							
Canseco 2021(53)	R	63.0	46/62	PEEK	108			1	9	73	26
		59.0	15/14	Titanium	29				2	24	3
Khan 2022(46)	P	63.5	42/72	PEEK	N/A						
		63.5	51/63	3D-Printed Ti	N/A						
Kim 2022(45)	R	58.53	21/22	PEEK	43	43 (DBM)			6	30	7
		59.17	21/19	3D-Printed Ti	40	40 (DBM)			4	28	8
Li 2020(54)	P	66.3	15/19	PEEK	34		1	1	1	21	10
		67.6	15/18	Allograft	33			1	1	21	10
Singhatanadgige 2022(55)	RCT	64.1	15/26	PEEK	50	50 (DBM)			7	25	18
		62.7	13/28	TiPEEK	49	49 (DBM)			7	37	5
Tanida 2016(56)	R	65	15/25	PEEK	51			2	4	30	15
		62.5	36/41	Titanium	93		1	7	14	54	17
Wu 2019(57)	P	55.3	76/97	PEEK	173		2	4	8	74	85
		54.2	92/114	Allograft	206			2	3	97	104

Authors	F/U length (mo.)	fusion assessment method	Fusion rate	VAS back Pre-op	VAS back Post-op	VAS leg Pre-op	VAS leg Post-op	ODI score Pre-op	ODI score Post-op
Cutler 2006	12	Xray	20/21					63	20.7
	12	Xray	18/18					63	22.8
Lv 2015	35	CT	79/84					42	21
	35	CT	91/96					45	19
Mura 2011	12	CT, dynamic Xray	56/56						
	12	CT, dynamic Xray	58/58						
Nemoto 2014	24	CT	22/23	6	1	5.6	0.6		
	24	CT	16/25	6.1	1.28	5.4	0.7		
Rickert 2017	12	CT, dynamic x-ray	20/22	7.2	3.6	6	3.7	42	27
	12	CT, dynamic x-ray	20/22	5.2	2.4	5.8	1.6	39	16
Vazifehdan 2019	50	x-ray, CT	296/323						
	50	x-ray, CT	91/96						
Canseco 2021	12	x-ray	100/108	7.2	2.9	7.1	1.8	43.2	22.0
	12	x-ray	25/29	7.5	4.2	7.8	2.0	40.0	18.0
Khan 2022	12	dynamic x-ray	83/114					45.2	29.5
	12	dynamic x-ray	102/114					43.4	24.9
Kim 2022	12	CT	42/43	2.0	2.3	6.0	3.0	45.6	36.9
	12	CT	38/40	4.4	2.0	6.3	2.9	50.8	38.4
Li 2020	24	dynamic x-ray	30/34					50.9	29.0
	24	dynamic x-ray	30/33					48.4	25.9
Singhatanadgige 2022	12	CT	45/50	6.3	1.4	7.3	1.7	48.2	12.7
	12	CT	47/49	6.6	1.5	6.6	0.9	53.5	12.4
Tanida 2016	24	CT, dynamic xray	41/51						
	24	CT, dynamic xray	77/93						
Wu 2019	24	dynamic x-ray	167/173					50.1	28.0
	24	dynamic x-ray	201/206					49.8	26.8

**Table 2:** Comparison of the different interbody materials.

Material	Male (n)	Female (n)	Local bone (n)	DBM (n)	HA + B-TCP	L1-2 (n)	L2-3 (n)	L3-4 (n)	L4-5 (n)	L5-S1 (n)	Fusion Segment (n)	Segment Level (n)	Fusion rate (%)
PEEK	299	401	662	111	20	3	9	45	339	138	993	1101	90.2 %
Ti	73	56	145	N/A	N/A	1	7	16	85	36	215	241	89.2 %
CFRP	N/A	N/A	N/A	N/A	N/A	N/A	N/A	N/A	N/A	N/A	58	58	100 %
Allograft	116	144	260	21	N/A	N/A	3	4	118	114	251	260	96.5 %
TiPEEK	19	42	71	49	22	N/A	1	16	51	5	67	71	94.4 %
Autograft	37	59	N/A	N/A	N/A	N/A	N/A	N/A	67	29	91	96	94.8 %
3D-Printed Ti	72	82	40	40	N/A	N/A	N/A	4	28	8	140	154	90.9 %

**Fig. 2:**
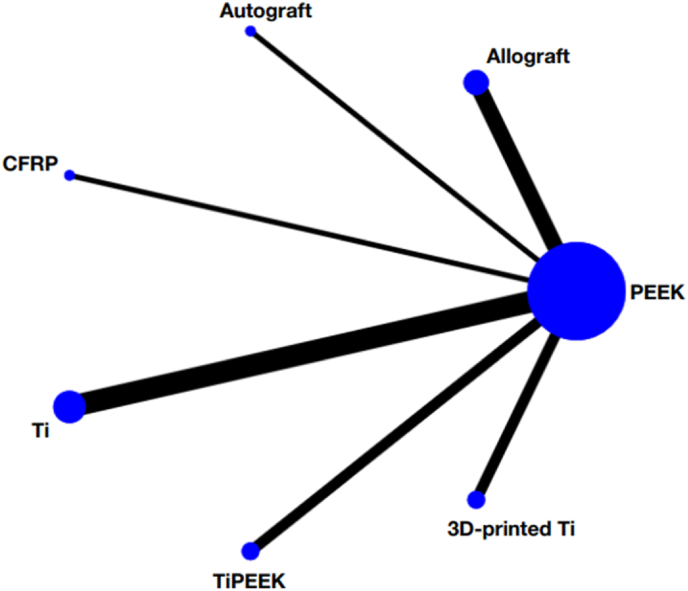
Network geometry of the consistency model of the different interbody materials. The size of each node is proportional to the total number of randomly assigned participants, and the width of each line is proportional to the number of studies comparing each pair of treatment strategies.

**Fig. 3:**
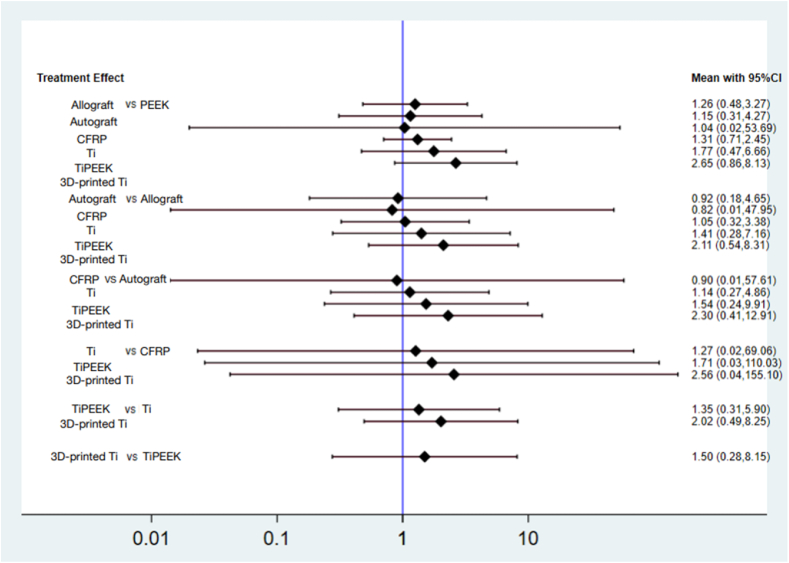
Interval plots of the different interbody materials.

Altogether were six types of head-to-head trials, which included PEEK vs. allograft (*n* = 106 patients), PEEK vs. autograft (*n* = 180), PEEK vs. CFRP (*n* = 100), PEEK vs. Ti (*n* = 721), PEEK vs. TiPEEK (*n* = 122) and PEEK vs. 3D-printed Ti (*n* = 311) (Fig. 2). A summary of the characteristics of each surgical approach including types of bone graft used, operated lumbar levels, lumbar fusion rates and functional outcomes, is presented in Table 2. The pooled results with PEEK material as the reference comprise the following: allograft (RR 1.26, 95 % CI 0.48 to 3.27), autograft (RR 1.15, 95 % CI 0.31 to 4.27), CFRP (RR 1.04, 95 % CI 0.02 to 53.69), Ti (RR 1.31, 95 % CI 0.71 to 2.45), TiPEEK (RR 1.77, 95 % CI 0.47 to 6.66) and 3D-printed Ti (RR 2.65, 95 % CI 0.86 to 8.13). Moreover, no statistically significant difference was found among the allograft, autograft, CFRP, Ti, TiPEEK and 3D-printed Ti (Fig. 3).

We performed the NMA with multiple treatment comparisons simultaneously in a single analysis including corresponding OR and 95 % CIs. The fusion assessment at the postoperative follow-up in the trials ranged between 12 and 50 months. We described the bias assessment using the Newcastle–Ottawa quality assessment form for nonrandomized study.^13^ Two of 13 studies were a prospective randomized controlled trial (RCT). Another 11 studies were assessed in the selection domain of which three studies demonstrated four stars, while eight studies demonstrated three stars. All 11 nonRCT studies demonstrated one star in the comparability domain and three stars in the outcome/exposure domain. Thus, eight studies received a total of seven stars and three studies received a total of eight stars. In other words, all studies revealed a low risk of bias (Table 3).

**Table 3:** The NewCastle-Ottawa scale (NOS) of the include studies.

Study	Selection	Comparability	Outcome/Exposure
Cutler 2016	★★★	★	★★★
Lv 2015	★★★	★	★★★
Mura 2011	★★★	★	★★★
Nemoto 2014	★★★	★	★★★
Vazifehdan 2019	★★★	★	★★★
Canseco 2021	★★★	★	★★★
Khan 2022	★★★★	★	★★★
Kim 2022	★★★	★	★★★
Li 2020	★★★★	★	★★★
Tanida 2016	★★★	★	★★★
Wu 2019	★★★★	★	★★★

**Fig. 4:**
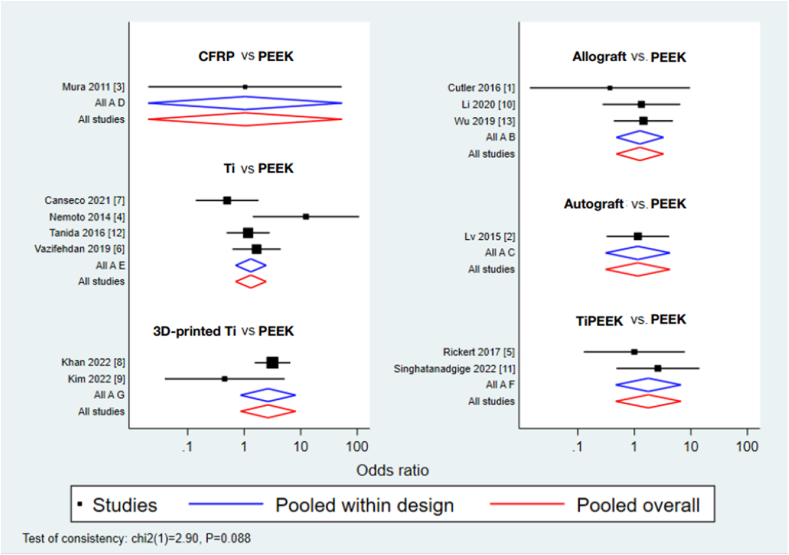
Network forest plot of the different interbody materials.

**Fig. 5:**
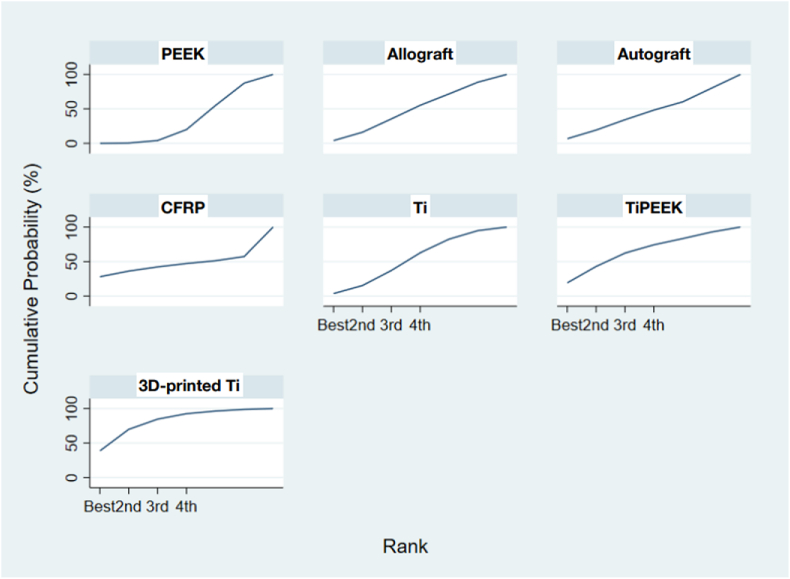
Results of the network rank test of the different interbody materials by the surface under the cumulative ranking curve (SUCRA) values.

The design-by-treatment interaction model did not show significant inconsistency in the NMA of the fusion rate (Chi-square = 2.90; *p* = 0.088; Fig. 4). These results were ranked based on the cumulative probability plots and the SUCRA values were determined (Fig. 5). Comparing PEEK with the other six materials, = no significant different rate of lumbar fusion was noted. The SUCRA scores of the seven materials revealed that 3D-printed Ti (SUCRA = 0.8) had the highest probabilities of being the best interbody material for fusion rates. The second and third rank belonged to TiPEEK (SUCRA = 0.6), Ti (SUCRA = 0.5) and allograft (SUCRA = 0.5), respectively. The fifth rank belonged to autograft (SUCRA = 0.4) and CFRP (SUCRA = 0.4). However, the opposite to rank cumulative probability, the SUCRA suggested that PEEK (SUCRA = 0.3) to be the material with the least success rate of fusion in TLIF.

## 4 Discussion

Lumbar interbody fusion plus posterior instrumentation has proved to provide superior fusion rates than posterolateral fusion alone.^16^^,^^17^ According to the TLIF approach, the unilateral working zone to perform discectomy and endplate preparation is narrow. As a result, TLIF accommodates a smaller interbody footprint compared with others.^18^ This would make a successful interbody fusion crucial because of the smaller interbody surface area. In addition to biocompatible and load carrying capacity, the ideal material interbody spacers should have 1) elastic modulus similar to cortical bone (18 GPa)^19^; 2) surface modification to improve bone on-growth and ingrowth^20^, ^21^, ^22^, ^23^; 3) radiolucency for easily assess radiographic fusion^19^ and 4) high level of support for osteogenic tissues.^24^

When compared Titanium (Ti) and PEEK, the commonly used interbody materials in clinical practice. Titanium has more favorable fusion rates than PEEK^24^^,^^25^ because of its high strength and elastic modulus which could potentially cause a stress shielding effect on the peri-implant bone and periprosthetic loosening.^26^ In vitro study demonstrated more human osteoblast differentiation and potential for improving cell adhesion on bone-titanium interface.^27^ PEEK is radiolucency allowing visualization of bone healing by normal radiographs and indicated less elastic modulus than cortical bone.^28^ Also, PEEK is hydrophobic in nature making it difficult to bond to bone. This lack of implant osteo-integration has been demonstrated in animal models.^25^

Hybrid technologies and surface coating have been made to combine the advantages of Ti and PEEK. TiPEEK composite with titanium endplate and a PEEK central portion demonstrated the osseo-integrative potential of titanium and radiolucency as well as favorable elastic modulus of PEEK.^29^ Walsh et al.^30^ demonstrated the histology of bone-implant interface of adult sheep comparing plasma-sprayed TiPEEK and PEEK implants at 4 and 12 weeks. The TiPEEK implants had direct bone ongrowth, whereas PEEK presented a fibrous union. Mechanical testing also confirmed at 4 and 12 weeks that hybrid implants had shear stress 8.0 and 18.1 MPa, while PEEK had 1.7 and 1.8 MPa, respectively. In term of subsidence rates, the titanium-coated PEEK also proved similar outcomes compared with the pre-existing modest subsidence of PEEK.^31^

CFRP PEEK had been developed and introduced during 1990(28). This material has the mechanical support, biocompatible and radiolucent properties as well as elastic modulus approximate to cortical bone.^32^^,^^33^ In addition, the material is compatible with CT and magnetic resonance imaging because of its nonmetal characteristics.^34^ Clinical studies have reported the interbody fusion success rates ranged from 70 to 100 %.^35^, ^36^, ^37^, ^38^ Although the fusion rates of CFRP are comparable with PEEK and Ti, the brittleness of carbon fiber constitutes a disadvantage.^33^ Tullberg reported the carbon cage could break if nonunion occurred.^39^ This could be the reason for the lower popularity of this material over time.

The structural allograft interbody provides an osteoconductive scaffold, which serves an essential biologic role to promote bone growth. Fatima et al.^12^ reported the structural allograft had 2.59-fold higher probability of fusion compared with PEEK. However, the subsidence rate of PEEK was lower than that of structural allografts. In term of cost effectiveness, related literature reported PEEK cages were more costly than structural allografts. The estimated cost for PEEK spacers were $4930 to $5246, whereas the structural allograft spacers were $1220 to $3640.^40^

The lamina, articular and spinous processes that were obtained during posterior decompression could be used as the morselized impacted bone grafts in the interbody space.^41^ Although this technique is less expensive, the increment in the disc height was significantly worse than using the cage (*p* < 0.012).^42^ It was explained in that the interbody cage is able to support the compression better than the morselized bone grafts.^42^ Autogenous iliac bone graft might be better in term of restore disc height; however, the method is not commonly used because of donor site pain and requiring more surgical intervention.^41^

3D-printed Ti is a recent development technology to improve osteo-integration of the cage by titrating the porosity, strut widths and orientation of surface modifications.^43^^,^^44^ McGilvray et al.^43^ demonstrated the histologic sections of bony ingrowth in ovine lumbar fusion model comparing PEEK, plasma sprayed porous titanium-coated PEEK (PSP) and 3D-printed Ti. PEEK implants were surrounded with poorly fibrous connective tissue, whereas the PSP implants had better neovascularization and slightly decreased fibrous union. The best osteo-integration was found in 3D-printed Ti, revealing increasing osteoblastic and osteoclastic remodeling as well as complete bony filling without fibrous union at the implant pores. Kim et al.^45^ reported no difference in terms of fusion rate between PEEK and 3D-printed Ti (95.0 % and 93.0 %) at 12 months. However, they found the quality or fusion grade I was better in the 3D-printed Ti than PEEK cage (37.5 vs. 16.3 % at 6 months, 77.5%vs. 51.2 % at 1 year). Furthermore, the subsidence rate of 3D-printed Ti indicates lower incidence than PEEK (23.5 vs. 40.2 %).^46^ The 3D-printed technology has a unique porous structure which could adjust the size of the porosity leading to maintaining the elastic modulus similar to the physiologic level.

The limitation in our review is that it only investigated studies using TLIF as the surgical approach without BMP application. Only 13 head-to-head studies with 1919 cases were included in our NMA. This small sample size may lead to underpowered pooled estimates and wide confidence intervals.

## 5 Conclusion

The NMA within the confines of our clinical study showed no significant difference in lumbar interbody fusion rate between materials of the TLIF interbody cage. However, 3D-printed titanium interbody cage statistically appeared to have the highest probability of being the best material for promoting fusion in TLIF. Future prospective comparative studies are required to confirm these conclusions and may help guide selecting the appropriate interbody material.

## CRediT authorship contribution statement

**Sutipat Pairojboriboon:** Writing – review & editing, Writing – original draft, Validation, Supervision, Project administration, Methodology, Investigation, Formal analysis, Conceptualization. **Supranee Niruthisard:** Writing – review & editing, Conceptualization. **Chandhanarat Chandhanayingyong:** Conceptualization. **Chalinee Monsereenusorn:** Conceptualization. **Siwaporn Poopan:** Methodology, Formal analysis, Data curation, Conceptualization. **Sheng-Fu Larry Lo:** Conceptualization, Methodology, Supervision.

## Declaration of competing interest

The authors declare that they have no known competing financial interests or personal relationships that could have appeared to influence the work reported in this paper.

## Abbreviations

TLIF: Transforaminal lumbar interbody fusion
PEEK: polyetheretherketone
CFRP: carbon fiber reinforcement to PEEK
Ti: titanium
NMA: network meta-analysis
PRISMA: Preferred Reporting Items for Systematic Reviews and Meta-Analyses
SP1: Sutipat Pairojboriboon
SP2: Siwaporn Poopan
SN: Supranee Niruthisard
BMP: bone morphogenic protein
CT: computed tomography
CI: confidence interval
OR: odds ratio
SUCRA: surface under the cumulative ranking
NOS: NewCastle-Ottawa Scale
RCT: randomized controlled trial
PSP: plasma sprayed porous titanium-coated PEEK
SD: standard deviation
GPa: Gigapascal
MRI: magnetic resonance imaging
DBM: demineralized bone matrix
F/U: follow up
mo: month
VAS: Visual Analogue Scale
ODI: Oswestry Disability Index
Pre-op: preoperative
Post-op: postoperative
3D: three dimensional
n: number
HA: hydroxyapatite
B-TCP: beta-tricalcium phosphate
n-HA/PA66: nanohydroxyapatite/polyamide-66
N/A: not available
